# Evaluation of Implementation and Effectiveness of China’s Antibiotic Stewardship in the First Affiliated Hospital of Sun Yat-sen University

**DOI:** 10.3390/antibiotics12040770

**Published:** 2023-04-17

**Authors:** Nianzhen Zheng, Jian Li, Yang Liu, Kang Liao, Jie Chen, Chengcheng Zhang, Weiping Wen

**Affiliations:** 1 Department of Otolaryngology, The First Affiliated Hospital of Sun Yat-sen University, Guangzhou Key Laboratory of Otorhinolaryngology, Otorhinolaryngology Institute of Sun Yat-sen University, Guangzhou 510080, China; 2 Information Data Center, The First Affiliated Hospital of Sun Yat-sen University, Guangzhou 510080, China; 3 Room of Microbiology, Department of Medical Laboratory, The First Affiliated Hospital of Sun Yat-sen University, Guangzhou 510080, China; 4 Department of Pharmacy, The First Affiliated Hospital of Sun Yat-sen University, Guangzhou 510080, China; 5 Department of Otolaryngology, The Sixth Affiliated Hospital of Sun Yat-sen University, Guangzhou 510655, China

**Keywords:** antibiotic stewardship, stewardship implementation, stewardship impact, China, evaluation research

## Abstract

Antibiotic stewardship has been prioritized by governments and health care organizations in recent years as antibiotic resistance is markedly increasing. A tertiary hospital in Guangzhou, China was chosen as a study example to undertake an implementation and effectiveness evaluation of China’s antibiotic stewardship to improve and promote antimicrobial stewardship nationwide. The general surgery department of the study hospital was utilized to examine surgical site infection, and samples from across the hospital were used to identify bloodstream infection. Data was analyzed using descriptive analysis, the Mann–Kendall trend test, logit model and panel data model, and *t*-tests. In terms of prophylactic and therapeutic antibiotic rational use, respectively, we evaluated implementation conditions, the correlation between implementation and corresponding disease progress, and the cost-effectiveness of China’s antibiotic stewardship. For perioperative prophylactic antibiotic use, antibiotic stewardship was found to have been well-implemented, cost-effective, and reduced the incidence of surgical site infection. However, concerning therapeutic use and antibiotic-resistant bacterial infection prophylaxis, the complexity of influencing factors and the contradiction between stewardship implementation and clinical demand needs to be further evaluated.

## 1. Introduction

Bacterial infection is a global issue, increasingly drawing the attention of healthcare workers, administrations, and organizations [1]. During several influenza pandemics that have occurred in the past century, pneumonia, caused by bacterial co-infection, was a main cause of mortality, accounting for 95% of deaths in the 1918 pandemic, 44% in the 1957–1958 pandemic, and approximately 20% (4–44%) of deaths in inter-pandemic years, according to research in the United States, England and Wales [2]. Secondary infection has also been a significant complication with COVID-19. A review published in December 2020 reported that 8% of patients with COVID-19 presented with bacterial/fungal coinfection at hospital admission, and 72% patients received antimicrobial therapy [3]. Antibiotic therapy is indispensable in the treatment of bacterial infection; however, antibiotic resistance caused by the misuse and overuse of antibiotics has become a serious global challenge.

Governments and health organizations worldwide have developed stewardship guidelines for the standardization of antibiotic use and to prevent drug-resistant bacterial infections. High-income countries’ antibiotic stewardship, mainly focus on: (1) education for healthcare providers concerning microbiology and antibiotics; (2) promotion of antibiotic stewardship in all health care settings; (3) education in antibiotic stewardship for the general public and healthcare providers; (4) establishment of surveillance systems and databases for antibiotic use and antibiotic resistance; and (5) promotion of further research in antibiotic resistance and stewardship [4,5,6]. However, in low- and middle-income countries, antibiotic stewardship is limited as a result of limitations in local healthcare systems [7]. To standardize the management of antimicrobial resistance, the World Health Organization (WHO) published the “Global Action Plan on Antimicrobial Resistance” in 2015. This document provides guidelines for governments to: (1) improve awareness and understanding of antimicrobial resistance through effective communication, education, and training; (2) strengthen knowledge and the evidence base through surveillance and research; (3) reduce the incidence of drug-resistant bacterial infection; (4) optimize the use of antimicrobial medicines; and (5) increase investment in new medicines, diagnostic tools, vaccines, and other interventions [1].

In China, the policy of improving the management of the clinical application of antibiotics was released by the General Office of the Bureau of Medical Administration and Hospital Authority in 2008. Since then, clinical antibiotic use in China has entered a new era of stewardship management. As opposed to antibiotic stewardship in most other countries, in China, national antibiotic stewardship is mandatory at all levels of the healthcare system. Antibiotic stewardship in China mainly involves three aspects: (1) guidelines and supervision regarding the use of antibiotics based on the rate of drug resistance; (2) regulation of prophylactic antibiotic use during the perioperative period; and (3) use of a drug grading system for the clinical use of antibiotics [8,9].

In the first aspect, the Ministry of Health of the People’s Republic of China has published several versions of the Guiding Principles for Clinical Application of Antibiotics and has developed monitoring systems for the rational use of antibiotics at all levels of the healthcare system.

The second aspect, the rational use of perioperative prophylactic antibiotics, concerns surgical procedures with clean incisions and clean-contaminated incisions. For clean incisions, antibiotics cannot be used during the perioperative period for prophylactic purposes, otherwise, it would be considered irrational antibiotic use. For clean-contaminated incisions, rational use of antibiotics can be separated into drug appropriateness and time appropriateness. Drug appropriateness refers to surgeons use of prophylactic antibiotics according to the common bacteria that cause particular site infections. For time appropriateness, preoperative antibiotic administration should begin 0.5–1 h before the start of surgery. Any antibiotics used during the perioperative period for prophylactic purposes beyond this regulation would be considered irrational use. Administration of postoperative antibiotics should be stopped within 24–48 h after surgery [10,11].

For the third aspect, the antibiotic grading system contains three grades. First is the unrestricted grade, which includes antibiotics that are cheap with good efficacy and fewer side effects, such as cefuroxime zinc and ceftriaxone. Unrestricted grade antibiotics can be prescribed by all-grade doctors. Second is the restricted grade, which contains antibiotics that have good efficacy but are expensive or have limitations regarding side effects or drug resistance, such as cefoperazone–sulbactam and piperacillin–tazobactam that can be prescribed by doctors more senior than resident doctors. Third are special-grade antibiotics, which have good efficacy but are expensive and work for special drug-resistant bacteria, and require usage restrictions for avoiding drug resistance production, such as imipenem, meropenem, and vancomycin, which can only be prescribed under the directions of both chief doctors and antibiotic experts [9].

This system of mandatory antimicrobial stewardship has been followed in China for more than 10 years and has been revised several times during this period. However, comprehensive evaluation is lacking to improve and promote antimicrobial stewardship. In comparison, evaluation reports in high-income countries mostly focus on the implementation of antimicrobial stewardship, its influencing factors, and the evaluation of cost and effectiveness [12,13,14]. In this study, we selected the First Affiliated Hospital of Sun Yat-sen University (FAH, SYSU) as our study site because, as a tertiary hospital, it has a complete antibiotics monitoring system and well-developed clinical electronic database in use since 2013. Thus, we selected a study period from 2013 to 2018 for the evaluation of antibiotic stewardship. This evaluation can provide insights into the effectiveness of China’s mandatory antimicrobial stewardship system and provide a reference for health administration departments to improve and promote antibiotic stewardship nationwide.

## 2. Results

### 2.1. Stewardship Implementation Evaluation

#### 2.1.1. Trend of Rational Use of Perioperative Prophylactic Antibiotics

**Figure 1:**
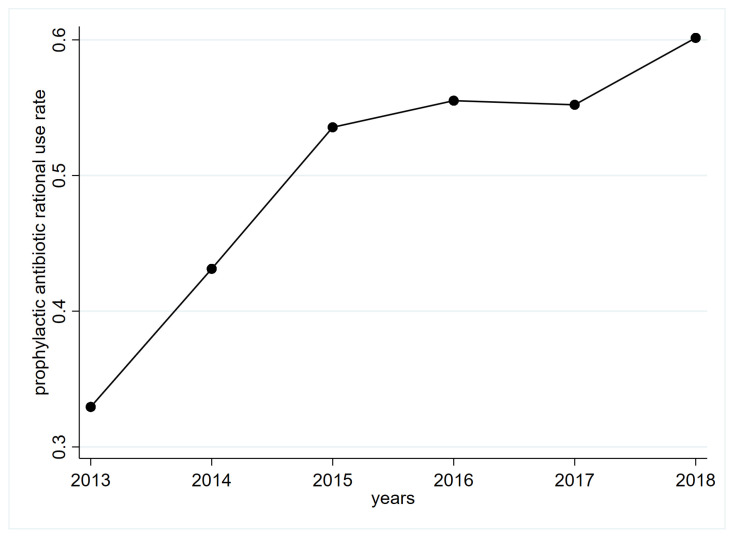
Trends of perioperative prophylactic antibiotic rational use rate in FAH, SYSU general surgery department. FAH, SYSU: First Affiliated Hospital of Sun Yat-sen University.

The rate of rational use perioperative prophylactic antibiotics in the general surgery department of FAH, SYSU increased each year, from 32.95% in 2013 to 60.19% in 2018. The increasing trend in the first three years was rapid and then slowed in subsequent years (Figure 1).

#### 2.1.2. Trends in the Detection Rate of Multiple Antibiotic-Resistant Bacteria and Drug Use Intensity for Each Grade of Antibiotics

**Figure 2:**
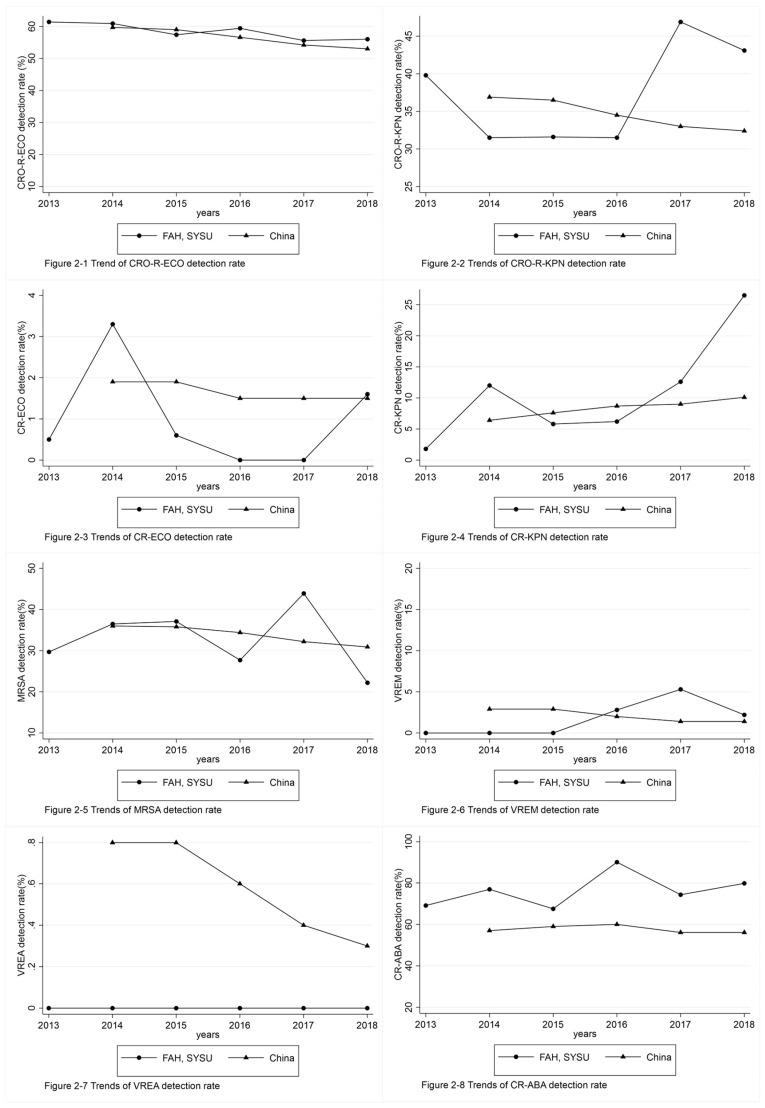
Comparison between the detection rate of multiple antibiotic-resistant bacteria among patients with bloodstream infection at FAH, SYSU from 2013 to 2018, and nationally reported detection rates for multiple antibiotic-resistant bacteria between 2014 and 2018. FAH, SYSU: First Affiliated Hospital of Sun Yat-sen University. CRO-R-ECO: ceftriaxone-resistant *Escherichia coli*, CRO-R-KPN: ceftriaxone-resistant *Klebsiella pneumoniae*, CR-ECO: carbapenem-resistant *Escherichia coli*, CR-KPN: carbapenem-resistant *Klebsiella pneumoniae*, MRSA: methicillin-resistant *Staphylococcus aureus*, VREM: vancomycin-resistant *Enterococcus faecium*, VREA: vancomycin-resistant *Enterococcus faecalis*, CR-ABA: *Acinetobacter baumannii*.

We assessed trends in the detection rate of multiple antibiotic-resistant bacteria among patients with bloodstream infection at FAH, SYSU from 2013 to 2018. A comparison with nationally reported trends in detection rates for multiple antibiotic-resistant bacteria between 2014 and 2018 is shown in Table 1 and Figure 2 [15,16,17,18,19]. During this timeframe, the detection rate of CRO-R-ECO decreased. The detection rate of IMP-R-KPN increased, while the detection rate of CRO-R-KPN, IMP-R-ECO, MRSA, VREM, VREA, and IMP-R-ABA were not statistically significant. As for the trends of nationally reported detection rates for multiple antibiotic-resistant bacteria between 2014 and 2018, the detection rate of third-generation cephalosporin-resistant *E. coli* (corresponding CRO-R-ECO in our sample), third-generation cephalosporin-resistant *Klebsiella pneumoniae* (corresponding CRO-R-KPN), MRSA, and VREA decreased significantly. The detection rate of carbapenem-resistant *Klebsiella pneumoniae* (CR-KPN, corresponding IMP-R-KPN) increased significantly. The detection rates of carbapenem-resistant E. coli (CR-ECO, corresponding IMP-R-ECO) and CR-ABA (corresponding IMP-R-ABA) demonstrated no significant changes.

**Figure 3:**
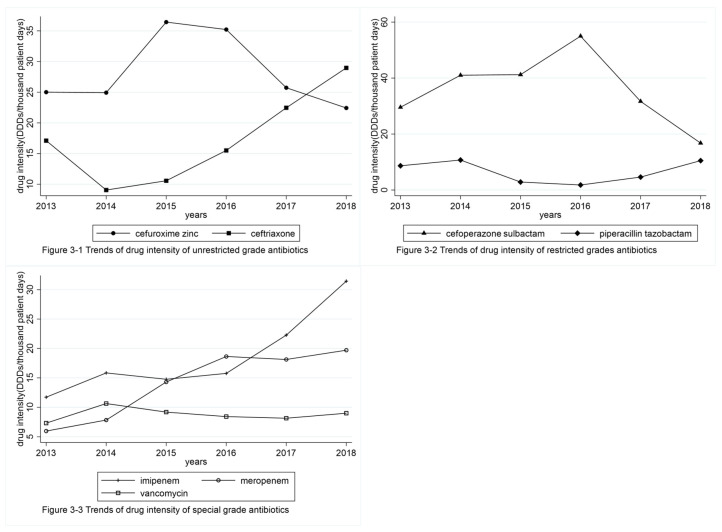
Trends of drug use intensity for each grade of antibiotics from 2013–2018 in FAH, SYSU. FAH, SYSU: First Affiliated Hospital of Sun Yat-sen University; DDDs: total dose of drug per year/defined daily dose of drug.

Trends in drug use intensity for each grade of antibiotics from 2013 to 2018 at FAH, SYSU are shown in Table 2 and Figure 3. Among unrestricted-grade antibiotics, the drug use intensity of cefuroxime zinc decreased, and the drug use intensity of ceftriaxone increased without a significant trend. Among restricted-grade antibiotics, the drug use intensity of cefoperazone–sulbactam and piperacillin–tazobactam demonstrated no significant changes. As for special-grade antibiotics, drug use intensity of both imipenem and meropenem increased, especially, imipenem, which increased in a significant trend, whereas that of vancomycin did not change significantly.

### 2.2. Relationship between Stewardship Implementation and Disease Progress

#### 2.2.1. Relativity Analysis of the Perioperative Prophylactic Antibiotic Use Aspect

**Figure 4:**
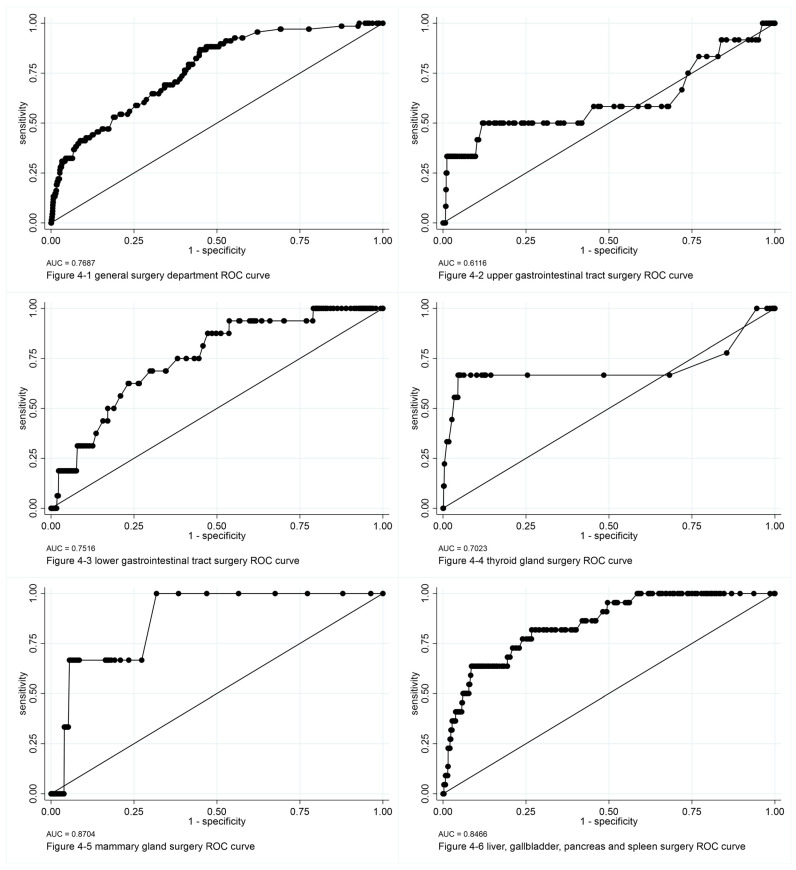
ROC curves of logit models of perioperative prophylactic antibiotic rational use analysis. ROC: receiving operator characteristic, AUC: area under the ROC curve.

From 2013 to 2018, the rate of surgical site infection in the general surgery department of FAH, SYSU decreased with an increased rate of rational perioperative prophylactic antibiotic use (Table 3, Figure 4).

After screening all variables with a logit model independently, we included the main study point of perioperative prophylactic antibiotic rational use, and smoking, alcohol consumption, and hospitalization duration in the logit model and panel model analysis as explanatory variables. In logit model analysis of the entire general surgery department, we found that in patients with rational prophylactic antibiotic use, the rate of surgical site infection was lower, and was 0.383 times that of patients with irrational prophylactic antibiotic use. We found that smoking and hospitalization duration were positively correlated with surgical site infection, whereas alcohol consumption and rational use of perioperative prophylactic antibiotics were negatively correlated with surgical site infection.

We also conducted a subgroup analysis according to surgical site. For clean-incision surgical sites, such as in surgeries of the thyroid or mammary gland, we found that rational use of perioperative prophylactic antibiotics was a main influencing factor that protected against surgical site infection. However, for surgical sites with clean-contaminated incisions, such as in surgeries of the upper and lower gastrointestinal tract, liver, and biliary system, the duration of hospitalization was the main influencing factor; longer hospitalization duration was related to higher rate of surgical site infection. As for panel model analysis, when considering the impact of time and surgical site, hospitalization duration was the only significant influencing factor.

#### 2.2.2. Relativity Analysis of Therapeutic Antibiotic Use Aspect

**Figure 5:**
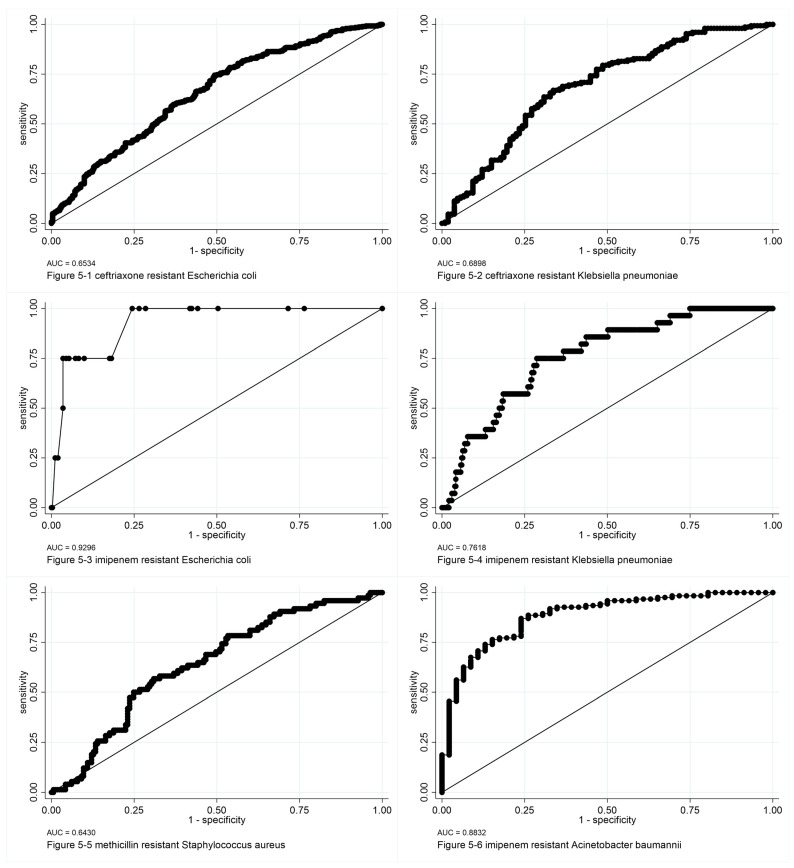
ROC curves of logit models of bacterial bloodstream infection analysis. ROC: receiving operator characteristic, AUC: area under the ROC curve.

From 2013 to 2018, the detection rate for multiple antibiotic-resistant bacteria in patients with bloodstream infection at FAH, SYSU decreased with a decreased ratio of antibiotic use duration to hospitalization duration. No significant correlation was found between the detection rate and drug use intensity of the corresponding antibiotics (Table 4 and Table 5, Figure 5).

In the logit model and the panel model analysis, the main study points include: total hospital drug use intensity; the ratio of antibiotic use duration/hospitalization duration; patient demographic characteristics; hospital department; and common influencing factors of bloodstream infection, such as neutrophil deficiency and vascular catheter use were included as variables in screening. Different variables were included in the analysis for different bacteria. In the logit model analysis, among all factors, the ratio of antibiotic use duration/hospitalization duration was the most common factor positively correlated with most bacteria investigated in this study. Regarding total hospital drug use intensity for the corresponding antibiotics, we found no relationship with the rate of detection of multiple antibiotic-resistant bacteria. Further panel model analysis confirmed this result.

### 2.3. Cost-Effectiveness Evaluation

#### 2.3.1. Cost-Effectiveness Evaluation of the Perioperative Prophylactic Antibiotic Use Aspect

The results of the cost comparison between perioperative prophylactic antibiotic rational use and irrational use are shown in Table 6. According to *t*-tests, patients with irrational antibiotic use had greater total and single day hospitalization costs, hospitalization medicine costs, and hospitalization antibiotics costs. On average, with rational antibiotic use per patient, CNY 578.33, 480.72, and 131.72 could be saved for the costs of single day hospitalization, medicines during hospitalization, and antibiotics during hospitalization, respectively, compared to one patient treated with irrational antibiotic use.

#### 2.3.2. Cost-Effectiveness Evaluation of Therapeutic Antibiotic Use Aspect

The results of cost comparisons between antibiotic use duration/hospitalization duration ratios ≤0.9 and >0.9 in patients with bacterial bloodstream infection is shown in Table 5. Except for total hospitalization cost, hospitalization medicine cost, and single day hospitalization cost in patients in VREA group and total hospitalization antibiotics costs in patients in VREM group, *t*-tests demonstrate that patients with an antibiotic use duration/hospitalization duration ratio ≤0.9 had lower total and single day hospitalization costs, hospitalization medicine costs, and hospitalization antibiotics costs per patient. Furthermore, in patients with an antibiotic use duration/hospitalization duration ratio ≤0.9, CNY 338.12–4203.06, CNY 380.63–1993.10, and CNY 329.25–1085.32 were saved in costs of single day hospitalization, and medicines and antibiotics given during hospitalization, respectively (Table 7).

## 3. Discussion

In this study, we aimed to comprehensively evaluate the implementation and influence of antibiotic stewardship in a tertiary hospital in China. Compared with studies globally, which have mostly focused on a single point, such as peri-operative prophylactic rational antibiotic use or rational antibiotic use for infectious disease treatment, this study evaluates both prophylactic and therapeutic antibiotic use in terms of implementation, relationship with disease progress, and cost-effectiveness. We found that the rate of prophylactic antibiotic rational use increased each year in the general surgery department of our study hospital, from 32.95% to 60.19%. Furthermore, our evaluation demonstrated that rational use of prophylactic antibiotics was a protective factor against surgical site infection, and patients with rational use of antibiotics had lower costs. A report of the United States Surgical Care Improvement Project evaluating effectiveness also identified that program compliance was related to incidence of surgical site infection [20]. However, both the average time-appropriate rate and average drug-appropriate rate were more than 85% in that report; results that might have been caused by analyzing time appropriateness and drug appropriateness separately.

As for rational therapeutic use of antibiotics, under the antibiotics grading system, the use intensity of representative drugs in each grade did not decrease significantly in our study. Even for imipenem and meropenem, which belong to the special grade that is most strictly controlled, their use intensity increased from 2013 to 2018. This indicates a contradiction between clinical demand and policy implementation. In the sample evaluation of bloodstream infection, compared with annual national antimicrobial resistance surveillance reports from 2014 to 2018, the isolation rates of antibiotic-resistant bacteria in FAH, SYSU were similar to the national rates. However, according to data from the European Centre for Disease Prevention and Control, the isolation rate of CRO-R-ECO is much lower than in China, with an increasing trend, and the isolation rate of VREM is much higher, with a similar trend [21,22,23,24,25,26]; which illustrates the differences between countries. Interestingly, in further analysis, we found that the isolation rate of the antibiotic-resistant bacteria investigated in this study was not related to the total hospital drug use intensity. Another factor related to drug use, the single-patient antibiotic use duration/hospitalization duration ratio, was positively related to the bacterial isolation rate. Moreover, costs were lower for patients with an antibiotic use duration/hospitalization duration ratio ≤0.9. The sample size and complexity of influencing factors in antibiotic resistance might limit the applicability of these findings, but our results indicate that improving antibiotic stewardship to better fit complex clinical conditions is important.

In current studies, outpatient rational use of antibiotics is a main focus of public health administrations. Currently, several large-scale antibiotic stewardship studies in high-income countries using nationally representative databases have reported their findings [27,28,29,30,31]. China’s public health research organization also published a nationwide report in early 2021 [32]. Although these were mostly observational studies concerning drug use, the findings serve as a reference regarding the direction of monitoring for health administration departments. Surgical prophylactic antibiotic rational use is another focus that is mainly a concern of clinical professionals. This kind of research mainly involves specific anatomical structures and related surgical site infection [33,34]. As for the relationship of antimicrobial stewardship implementation and compliance with surgical site infection, the only national-level research has been conducted in the United States [20]. Although a system of antimicrobial stewardship and antimicrobial resistance monitoring has been established in China, similar to those in many high-income countries, reports on the connection between observed monitoring data and clinical research into infection owing to antibiotic-resistant bacteria remain rare in China [35,36].

In this study, we focused on a tertiary hospital and used general surgery and bloodstream infection as samples to combine monitoring and clinical data in a comprehensive evaluation, including the implementation, effectiveness, and cost-effectiveness of antibiotic stewardship. Our results can provide a reference for improving antimicrobial stewardship and serve as an evaluation model for other medical institutions and government health departments.

As an experimental sample analysis, our study has some limitations. First, the time period of the study was from 2013 to 2018. Because antibiotic stewardship in China was initiated in 2008, we cannot make comparisons with the period prior to 2008. Second, apart from monitoring data of the hospital laboratory examination system, our data were derived from the hospital medical records, which are collected by thousands of clinical staff; thus, the subjectivity involved in the data derived from medical records cannot be ignored. Third, because the clinical medical record system at our study site was incomplete, outpatient data were not included in this study. Fourth, compliance with antibiotic stewardship in our large tertiary study hospital cannot be representative of stewardship in secondary and primary medical institutions, where problems of antibiotic overuse and misuse are significantly more serious. Nor can this sample evaluation research be considered to be the fully representative of the implementation and effectiveness of national antibiotic stewardship across China.

## 4. Materials and Methods

This study was a retrospective observational sample evaluation focused on FAH, SYSU during the period 2013–2018. This evaluation comprised three parts: (1) evaluation of antibiotic stewardship implementation; (2) evaluation of the relationship between antibiotic stewardship implementation and disease progress; and (3) evaluation of cost-effectiveness. According to the main contents of China’s antibiotic stewardship, we focused on two aspects in each of the above parts of the study: rational use of prophylactic antibiotics during the perioperative period and antibiotic rational use in the treatment of infectious disease. Considering the integrity of medical records in FAH, SYSU, surgical site infection in the general surgery department was chosen as the sample for evaluation of perioperative prophylactic antibiotic use. As a type of hospital-acquired infection in sterile tissue, we chose bloodstream infection to investigate the rational use of antibiotics for infectious disease treatment. Among the seven most common multidrug-resistant bacteria in China, we included methicillin-resistant *Staphylococcus aureus* (MRSA); extended-spectrum β-lactamase-producing gram-negative bacteria (ESBLs), including ceftriaxone-resistant *Escherichia coli* (CRO-R-ECO) and ceftriaxone-resistant *Klebsiella pneumoniae* (CRO-R-KPN); vancomycin-resistant enterococci, including vancomycin-resistant *Enterococcus faecium* (VREM) and vancomycin-resistant *Enterococcus faecalis* (VREA); carbapenem-resistant *Enterobacteriaceae,* including imipenem-resistant *E. coli* (IMP-R-ECO) and imipenem-resistant *Klebsiella pneumoniae* (IMP-R-KPN); and carbapenem-resistant *Acinetobacter baumannii* (CR-ABA), including imipenem-resistant *Acinetobacter baumannii* (IMP-R-ABA). [37]

### 4.1. Sample Selection

**Figure 6:**
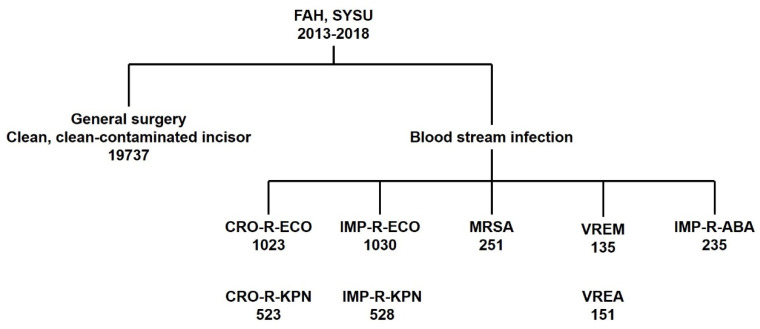
Patient inclusion chart.

After exclusion of patients with missing data, study inclusion patients are shown in Figure 6. In the evaluation of perioperative prophylactic antibiotic rational use, we included 19,373 patients admitted to the general surgery department of FAH, SYSU from 2013 to 2018 who underwent surgeries involving a clean or clean-contaminated incision. For the rational use of antibiotics in the treatment of the patients finally processed for bloodstream infection, we included patients who were admitted to all departments of FAH, SYSU. Among the total, 1023 patients were included in the CRO-R-ECO group, 523 patients in the CRO-R-KPN group, 1030 patients in the IMP-R-ECO group, 528 patients in the IMP-R-KPN group, 251 patients in the MRSA group, 135 patients in the VREM group, 151 patients in the VREA group, and 235 patients were included in the IMP-R-ABA group. Each group contained patients infected by multiple antibiotic-resistant bacteria and their corresponding antibiotic-sensitive bacteria.

### 4.2. Evaluation Plan

As mentioned, this study comprised three parts. First, in the evaluation of antibiotic stewardship implementation, we investigated the rational use of perioperative prophylactic antibiotics, the monitoring of multiple antibiotic-resistant bacteria, and the use of common antibiotics. In examining the relationship between stewardship implementation and disease progress, we focused on perioperative prophylactic antibiotic rational use and surgical site infection, as well as clinical use of antibiotics and bloodstream infection owing to multiple antibiotic-resistant bacteria. Finally, we conducted a cost-effectiveness evaluation in terms of perioperative prophylactic antibiotic rational use, as well as the rational use of antibiotics in patients with a bloodstream infection.

### 4.3. Data Collection

Most data were collected from the electronic clinical database in the information data center of FAH, SYSU. We included the clinical data of patients admitted into the general surgery department, hospitalized patients with a bloodstream infection caused by multiple antibiotic-resistant bacteria or the corresponding antibiotic-sensitive bacteria, and data of antibiotic-use intensity (DDDs/thousand patient days, DDDs: total dose of drug per year/defined daily dose of drug) in each hospital department at each antibiotic grade of FAH, SYSU between 2013 and 2018. Detection data of antibiotic-resistant bacteria were collected from the monitoring system in the microbiology unit of the medical laboratory department.

### 4.4. Data Integration and Analysis

After formatting the original data, all data were sorted into binary, categorical, and continuous variables. Among these variables, in the evaluation of perioperative prophylactic antibiotic rational use, we chose the main study point, perioperative prophylactic antibiotic use, as well as patients’ demographic characteristics, the common influencing factors for surgical site infection, such as smoking, alcohol consumption, and duration of hospitalization, as the screening explanatory variables. As for the rational use of antibiotics in the treatment of the patients with bloodstream infection, we chose the main study point, total hospital drug use intensity and the ratio of antibiotics use duration/hospitalization duration, as well as patients’ demographic characteristics, department in which patient is hospitalized, and common influencing factors of bloodstream infection, such as neutrophil deficiency and vascular catheter use, as the screening explanatory variables. For data analysis, descriptive analysis and Mann–Kendall trend tests were used to evaluate antibiotic stewardship implementation. We used a logit model and a panel data model to evaluate the relationship between stewardship implementation and disease progress. In this area, we carried out subgroup analysis in a logit model analysis of relativity of the perioperative prophylactic antibiotic use. The subgroup was categorized by different surgical sites, such as surgeries of the thyroid gland, mammary gland, the upper and lower gastrointestinal tract, liver, and biliary system. We conducted the cost-effectiveness analysis using *t*-tests. Data integration and data analysis were performed using Stata SE 15.1 for Mac (StataCorp LLC, College Station, TX, USA).

## 5. Conclusions

Our findings indicate that at FAH, SYSU, antibiotic stewardship for perioperative prophylactic antibiotic use was effectively implemented, reduced the incidence of surgical site infection, and lowered costs. However, for therapeutic antibiotic use and infection prophylaxis for antibiotic-resistant bacteria, the complexity of influencing factors and contradiction between stewardship implementation and clinical demand should be further evaluated. Our evaluation can serve as a reference for other healthcare organizations in future large-scale studies of antimicrobial stewardship implementation, effectiveness, and assessment.

## Figures and Tables

**Table 1:** Comparison between trends in the detection rate of multiple antibiotic-resistant bacteria among patients with bloodstream infection at FAH, SYSU from 2013 to 2018, and nationally reported trends in detection rates for multiple antibiotic-resistant bacteria between 2014 and 2018.

Multi-Drug Resistant Bacteria	Nation (Kendall’s Score (Prob > |z|))	FAH, SYSU (Kendall’s Score (Prob > |z|))
**CRO-R-ECO**	−10 (0.03)	−11 (0.06)
**CRO-R-KPN**	−10 (0.03)	4 (0.57)
**CR-ECO**	−6 (0.15)	−2 (0.85)
**CR-KPN**	10 (0.03)	11 (0.06)
**MRSA**	−10 (0.03)	−1 (1.00)
**VREM**	−8 (0.07)	8 (0.16)
**VREA**	−9 (0.04)	0 (1.00)
**CR-ABA**	−3 (0.61)	5 (0.45)

**Table 2:** Trends of drug use intensity for each grade antibiotics from 2013–2018 in FAH, SYSU.

Antibiotics	Kendall’s Score (Prob > |z|)
**Cefuroxime zinc**	−3 (0.71)
**Ceftriaxone**	9 (0.13)
**Cefoperazone sulbactam**	−1 (1)
**Piperacillin tazobactam**	−1 (1)
**Imipenem**	11 (0.06)
**Meropenem**	13 (0.02)
**vancomycin**	−1 (1.00)

**Table 3:** Logit model and panel data analyses of perioperative prophylactic antibiotic rational use in the general surgery department and surgical-site sub-analysis.

Factors	General Surgery	Upper Gastrointestinal Tract	Lower Gastrointestinal Tract	Thyroid Gland	Mammary Gland	Liver, Gallbladder, Pancreas and Spleen	Panel Data
Smoking	2.010 * (0.752) [0.966;4.183]	1.069 (1.148) [0.130;8.777]	2.286 (1.505) [0.630;8.304]	-	5.075 (6.686) [0.384;67.123]	2.870 * (1.747) [0.870;9.464]	0.266 (0.277) [−0.411;0.943]
Alcohol consumption	0.406 ** (0.179) [0.172;0.961]	0.732 (0.851) [0.075;7.151]	0.105 * (0.122) [0.011;1.022]	1.647 (1.772) [0.200;13.567]	-	0.526 (0.343) [0.146;1.888]	−0.255 (0.299) [−0.987;0.476]
Hospitalization duration	0.041 *** (0.006) [0.031;0.052]	0.056 *** (0.016) [0.025;0.088]	0.040 *** (0.010) [0.021;0.059]	−0.034 (0.077) [−0.184;0.117]	0.037 (0.182) [−0.320;0.394]	0.050 *** (0.008) [0.035;0.065]	0.0002 ** (0.0001) [0.0001;0.0003]
Prophylactic antibiotic rational use	0.383 *** (0.128) [0.199;0.739]	-	0.674(0.536) [0.141;3.209]	0.025 *** (0.018) [0.006;0.106]	0.084 * (0.120) [0.005;1.395]	0.781 (0.465) [0.243;2.508]	0.005 (0.004) [−0.004;0.014]
Cons	0.002 *** (0.001) [0.002;0.004]	0.002 *** (0.001) [0.001;0.008]	0.002 *** (0.001) [0.001;0.005]	0.030 *** (0.026) [0.006;0.161]	0.005 *** (0.010) [0.000;0.209]	0.001 *** (0.001) [0.000;0.003]	−0.013 * (0.006) [−0.027;0.002]
AUC	0.769	0.612	0.752	0.702	0.807	0.847	0.283 (rho)

The data reporting format is as follows. Every cell has two lines of data. In logit model, the first line shows OR (for binary variable) or correlation coefficient (for continuous variable)* (standard error); In the panel model: correlation coefficient* (standard error). The second line means [95% confidence interval]. AUC: area under the curve. * Significant at 5% level. ** Significant at 1% level. *** Significant at 0.1% level.

**Table 4:** Results of logit model analysis for bacterial bloodstream infection.

Factors	CRO-R-ECO	CRO-R-KPN	IMP-R-ECO	IMP-R-KPN	MRSA	IMP-R-ABA
**Age**						
≤14 y	-	-	-	-	-	-
14 < y ≤ 40	-	-	14.613 ** (18.528) [1.218;175.380]	-	-	-
40 < y ≤ 60	-	-	-	1.161 (0.497) [0.501;2.689]	0.607 (0.218) [0.300;1.229]	-
>60 y	-	-	-	-	-	-
**Gender**	-		0.297 (0.350) [0.030;2.983]	-	0.727 (0.212) [0.410;1.288]	1.959 (0.894) [0.801;4.794]
**Neutrophil** **deficiency**	0.799 (0.243) [0.440;1.451]		24.121 ** (37.794) [1.119;520.131]	-	-	-
**Vascular** **catheter**	-	0.938 (0.263) [0.541;1.627]	-	1.736 (0.779) [0.720;4.185]	-	3.362 ** (2.035) [1.027;11.009]
**Department**						
Medicine	0.404 *** (0.091) [0.261;0.627]	0.409 ** (0.153) [0.196;0.854]	1.155 (1.733) [0.061;21.878]	-	-	3.915 ** (2.590) [1.071;14.314]
Surgery	-	-	21.852 * (36.059) [0.861;554.718]	3.616 *** (1.698) [1.441;9.076]	-	-
Gynecology and obstetrics	4.880 *** (2.800) [1.585;15.023]	-	-	-	-	-
Pediatrics	0.268 *** (0.101) [0.128;0.563]	0.121 *** (0.057) [0.048;0.304]	-	-	-	-
Neurology	-	-	-	-	-	-
ICU	0.627 * (0.151) [0.392;1.004]	-	-	3.820 ** (2.166) [1.257;11.607]	-	5.534 ** (3.970) [1.356;22.580]
**Antibiotic use duration/** **hospitalization duration**	1.458 *** (0.356) [0.760;2.155]	1.161 * (0.669) [−0.151;2.471]	-	2.616 ** (1.919) [0.282;4.950]	1.539 *** (0.564) [0.433;2.645]	6.924 *** (1.271) [4.433;9.414]
**Cons**	0.624 * (0.168) [0.368;1.058]	0.881 (0.376) [0.382;2.034]	0.000 *** (0.001) [4.55;0.015]	0.003 *** (0.003) [0.000;0.022]	0.196 *** (0.098) [0.073;0.524]	0.001 *** (0.002) [0.000;0.015]
**AUC**	0.653	0.690	0.930	0.762	0.643	0.883

The data reporting format is as follows. The first line: OR (for binary variable) or correlation coefficient (for continuous variable)* (standard error). * Significant at 5% level. ** Significant at 1% level. *** Significant at 0.1% level. The second line: [95% confidence interval]. AUC: area under the curve, ICU: intensive care unit, CRO-R-ECO: ceftriaxone-resistant *Escherichia coli*, CRO-R-KPN: ceftriaxone-resistant *Klebsiella pneumoniae*, IMP-R-ECO: imipenem-resistant *Escherichia coli*, IMP-R-KPN: imipenem-resistant *Klebsiella pneumoniae*, MRSA: methicillin-resistant *Staphylococcus aureus*, IMP-R-ABA: imipenem-resistant *Acinetobacter baumannii*.

**Table 5:** Results of panel model analysis for bacterial bloodstream infection.

Factors	CRO-R-ECO	CRO-R-KPN	IMP-R-ECO	IMP-R-KPN	MRSA	IMP-R-ABA
**Age**						
≤14 y	-	-	-	-	-	-
14 < y ≤ 40	-	-	−0.001 (0.025) [−0.062;0.061]	-	-	-
40 < y ≤ 60	-	−0.397 (0.337) [−1.221;0.426]	-	-	-	-
>60 y	-	-	-	-	-	-
**Gender**	-	-	0.009 (0.010) [−0.016;0.035]	0.078 (0.099) [−0.164;0.320]	−0.142 (0.185) [−0.596;0.311]	-
**Neutrophil** **deficiency**	0.624 (0.449) [−0.475;1.723]	-	0.010 (0.021) [−0.041;0.061]	-	−0.621 *** (0.093) [−0.850;−0.392]	-
**Vascular catheter**	-	−0.532 (0.367) [−1.430;0.367]	-	0.296 *** (0.060) [0.150;0.442]	-	−0.265 * (0.124) [−0.568;0.039]
**antibiotic use duration/hospitalization duration**	0.904 ** (0.288) [0.199;1.608]	0.716 * (0.346) [−0.132;1.563]	-	0.491 *** (0.106) [0.232;0.749]	−0.153 (0.162) [−0.548;0.243]	0.576 ** (0.199) [0.088;1.064]
**Drug intensity**	0.000 (0.004) [−0.009;0.010]	0.021 ** (0.008) [0.001;0.042]	0.000 (0.000) [−0.001;0.001]	0.003 (0.002) [−0.002;0.007]	-	0.003 (0.002) [−0.003;0.009]
**cons**	−0.127 (0.264) [−0.773;0.519]	−0.134 (0.332) [−0.946;0.678]	−0.003 (0.020) [−0.051;0.045]	−0.575 *** (0.149) [−0.941;−0.210]	0.559 *** (0.103) [0.307;0.811]	0.122 (0.196) [−0.357;0.601]
**rho**	0.824	0.877	0.263	0.445	0.670	0.611

The data reporting format is as follows. The first line: correlation coefficient* (standard error). * Significant at 5% level. ** Significant at 1% level. *** Significant at 0.1% level. The second line: [95% confidence interval]. CRO-R-ECO: ceftriaxone-resistant *Escherichia coli*, CRO-R-KPN: ceftriaxone-resistant *Klebsiella pneumoniae*, IMP-R-ECO: imipenem-resistant *Escherichia coli*, IMP-R-KPN: imipenem-resistant *Klebsiella pneumoniae*, MRSA: methicillin-resistant *Staphylococcus aureus*, IMP-R-ABA: imipenem-resistant *Acinetobacter baumannii*.

**Table 6:** Results of *t*-tests for cost comparisons between rational and irrational use of prophylactic antibiotics.

	Irrational Use (CNY) Mean (SD)	Rational Use (CNY) Mean (SD)	Difference Mean (95% Confidence Interval)	*p* Value
Total hospitalization cost	65,699 (43,533)	19,925 (15,782)	45,774 (44,886; 46,662)	<0.0001
Total hospitalization medicine cost	17,531 (19,722)	3135 (4121)	14,396 (14,012; 14,780)	<0.0001
Total hospitalization antibiotics cost	2891 (7637)	24 (113)	2867 (2722; 3012)	<0.0001
Single day hospitalization cost	3957.36 (1875.04)	3379.02 (1775.91)	578.33 (527.32; 629.34)	<0.0001
Single day hospitalization medicine cost	912.58 (517.44)	431.86 (382.07)	480.72 (468.14; 493.30)	<0.0001
Single day hospitalization antibiotics cost	133.62 (181.52)	1.90 (8.13)	131.72 (128.26; 135.17)	<0.0001

SD: standard deviation.

**Table 7:** Cost comparisons (*t*-tests) for antibiotic use duration/hospitalization duration ratio ≤0.9 and >0.9 in bloodstream infection with multiple antibiotic-resistant bacteria and their corresponding antibiotic-sensitive bacteria.

	Antibiotic Use Duration/Hospital Duration Ratio ≤ 0.9 (CNY) Mean (SD)	Antibiotic Use Duration/Hospital Duration Ratio > 0.9 (CNY) Mean (SD)	Difference Mean (95% Confidence Interval)	*p* Value
**CRO-R-ECO**				
Total hospitalization cost	65,653 (77,915)	86,712 (123,731)	−21,059 (−34,475; −7643)	<0.0001
Total hospitalization medicine cost	27,517 (35,559)	41,276 (61,377)	−13,758.67 (−20,162; −7355)	<0.0001
Total hospitalization antibiotic cost	10,509 (16,477)	19,570 (30,384)	−9060 (−12,142; −5978)	<0.0001
Single day hospitalization cost	3065.13 (2592.14)	3506.32 (3278.07)	−441.19 (−843.14; −39.24)	0.02
Single day hospitalization medicine cost	1228.17 (969.27)	1608.80 (1502.40)	−380.63 (−545.48; −215.78)	<0.0001
Single day hospitalization antibiotic cost	473.18 (545.09)	804.22 (865.28)	−331.03 (−424.88; −237.19)	<0.0001
**CRO-R-KPN**				
Total hospitalization cost	73,121 (85,839)	166,851 (297,954)	−93,731 (−130,967; −56,494)	<0.0001
Total hospitalization medicine cost	30,305 (37,246)	75,764 (116,954)	−45,459 (−60,266; −30,653)	<0.0001
Total hospitalization antibiotic cost	11,794 (17,363)	35,756 (54,275)	−23,961 (−30,837; −17,086)	<0.0001
Single day hospitalization cost	3062.93 (2604.67)	4332.79 (3626.66)	−1269.87 (−1845.96; −693.77)	<0.0001
Single day hospitalization medicine cost	1232.37 (1004.32)	2075.69 (1760.20)	−843.32 (−1098.44; −588.20)	<0.0001
Single day hospitalization antibiotic cost	469.10 (514.17)	1052.24 (1069.62)	−583.14 (−730.21; −436.06)	<0.0001
**IMP-R-ECO**				
Total hospitalization cost	65,347 (77,695)	86,492 (123,554)	−21,145 (−34,494; −7796)	0.001
Total hospitalization medicine cost	27,410 (35,457)	41,174 (61,285)	−13,764 (−20,135; −7394)	<0.0001
Total hospitalization antibiotic cost	10,497 (16,436)	19,527 (30,335)	−9030 (−12,096; −5963)	<0.0001
Single day hospitalization cost	3059.62 (2583.40)	3503.42 (3272.32)	−443.80 (−843.58; −44.03)	0.02
Single day hospitalization medicine cost	1227.11 (967.81)	1608.01 (1499.66)	−380.90 (−545.00; −216.81)	<0.0001
Single day hospitalization antibiotic cost	474.95 (546.00)	804.20 (863.67)	−329.25 (−422.81; −235.70)	<0.0001
**IMP-R-KPN**				
Total hospitalization cost	79,178 (100,321)	190,897 (315,547)	−111,719 (−151,646; −71,792)	<0.0001
Total hospitalization medicine cost	32,818 (42,188)	82,902 (118,860)	−50,084 (−65,368; −34,799)	<0.0001
Total hospitalization antibiotic cost	12,689 (20,631)	39,357 (54,741)	−26,668 (−33,779; −19,557)	<0.0001
Single day hospitalization cost	3150.03 (2831.38)	4812.55 (5217.51)	−1662.51 (−2402.13; −922.90)	<0.0001
Single day hospitalization medicine cost	1272.74 (1091.30)	2190.84 (1959.10)	−918.10 (−1198.11; −638.09)	<0.0001
Single day hospitalization antibiotic cost	471.53 (558.86)	1124.68 (1132.59)	−653.15 (−809.34; −496.96)	<0.0001
**MRSA**				
Total hospitalization cost	68,243 (72,103)	107,927 (197,530)	−39,684 (−75,445; −3923)	0.02
Total hospitalization medicine cost	25,402 (28,616)	41,266 (53,740)	−15,864 (−26,675; −5053)	0.002
Total hospitalization antibiotic cost	10,512 (12,875)	22,374 (32,649)	−11,862 (−178,75; −5849)	<0.0001
Single day hospitalization cost	2509.47 (1756.42)	3723.29 (3331.72)	−1213.82 (−1881.69; −545.95)	<0.0001
Single day hospitalization medicine cost	917.60 (793.15)	1553.30 (1399.23)	−635.70 (−923.84; −347.56)	<0.0001
Single day hospitalization antibiotic cost	386.92 (411.82)	839.39 (778.64)	−452.47 (−608.73; −296.20)	<0.0001
**VREM**				
Total hospitalization cost	211,330 (167,709)	409,278 (468,115)	−197,948 (−351,037; −44,859)	0.01
Total hospitalization medicine cost	96,919 (90,312)	147,169 (162,523)	−50,250 (−107,671; 7170)	0.04
Total hospitalization antibiotic cost	40,767 (48,212)	59,252 (72,928)	−18,486 (−45,531; 8560)	0.09
Single day hospitalization cost	5948.38 (4003.43)	10,567.25 (7376.56)	−4618.87 (−7210.29; −2027.45)	<0.0001
Single day hospitalization medicine cost	2585.94 (1604.79)	3852.01 (2493.40)	−1266.07 (−2183.13; −349.00)	0.004
Single day hospitalization antibiotic cost	1021.59 (753.17)	1549.26 (1096.27)	−527.66 (−939.31; −116.02)	0.01
**VREA**				
Total hospitalization cost	114,734 (184,394)	116,242 (104,842)	−1507 (−66,039; 63,025)	0.48
Total hospitalization medicine cost	39,185 (55,355)	50,977 (45,812)	−11,792 (−32,741; 9158)	0.13
Total hospitalization antibiotic cost	15,245 (27,463)	24,457 (26,432)	−9212 (−20,097; 1672)	0.05
Single day hospitalization cost	3508.72 (2596.19)	3846.84 (3506.75)	−338.12 (−1523.96; 847.73)	0.29
Single day hospitalization medicine cost	1218.13 (913.97)	1765.10 (1841.76)	−546.97 (−1077.52; −16.41)	0.02
Single day hospitalization antibiotic cost	462.76 (558.10)	896.62 (1229.68)	−433.87 (−779.05; −88.68)	0.01
**IMP-R-ABA**				
Total hospitalization cost	200,010 (312,307)	365,231 (355,864)	−165,221 (−263,829; −66,612)	0.001
Total hospitalization medicine cost	71,400 (106,936)	146,460 (119,965)	−75,060 (−108,489; −41,630)	<0.0001
Total hospitalization antibiotic cost	29,174 (48,533)	65,653 (51,239)	−36,479 (−51,089; −21,868)	<0.0001
Single day hospitalization cost	6427.34 (5491.09)	10,630.40 (8916.52)	−4203.06 (−6438.45; −1967.66)	<0.0001
Single day hospitalization medicine cost	2289.16 (2176.96)	4282.26 (2886.75)	−1993.10 (−2754.69; −1231.51)	<0.0001
Single day hospitalization antibiotic cost	950.40 (1158.17)	2035.71 (1552.00)	−1085.32 (−1493.53; −677.10)	<0.0001

SD: standard deviation; CRO-R-ECO: ceftriaxone-resistant *Escherichia coli*, CRO-R-KPN: ceftriaxone-resistant *Klebsiella pneumoniae*, IMP-R-ECO: imipenem-resistant *Escherichia coli*, IMP-R-KPN: imipenem-resistant *Klebsiella pneumoniae*, MRSA: methicillin-resistant *Staphylococcus aureus*, VREM: vancomycin-resistant *Enterococcus faecium*, VREA: vancomycin-resistant *Enterococcus faecalis*, IMP-R-ABA: imipenem-resistant Acinetobacter baumannii.

## Data Availability

The datasets used and/or analyzed during the current study are available from the corresponding author on request.

## Abbreviations

FAH, SYSU: the First Affiliated Hospital of Sun Yat-sen University; MRSA: methicillin resistant *Staphylococcus aureus*; ESBLs: extended spectrum β-lactamases producing Gram-negative bacteria; VRE: vancomycin-resistant *Enterococci*; CRE: carbapenem-resistant *Enterobacteria*; CR-ABA: carbapenem-resistant *Acinetobacter baumannii*; CRO-R-ECO: ceftriaxone resistant *Escherichia coli*; CRO-R-KPN: ceftriaxone resistant *Klebsiella pneumoniae*; IMP-R-ECO: imipenem resistant *Escherichia coli*; IMP-R-KPN: imipenem resistant *Klebsiella pneumoniae*; VREM: vancomycin resistant *Enterococcus faecium*; VREA: vancomycin resistant *Enterococcus faecalis*; IMP-R-ABA: imipenem resistant *Acinetobacter baumannii*; CR-ECO: carbapenem-resistant *Escherichia coli*; CR-KPN: carbapenem-resistant *Klebsiella pneumoniae*.
